# Relationship between Consulting for Second Medical Opinions, Radiotherapy, and Satisfaction with Therapy, Analyzed by Structural Equation Modeling: A Web-Based Survey

**DOI:** 10.31557/APJCP.2021.22.9.2889

**Published:** 2021-09

**Authors:** Masanari Minamitani, Tomoya Mukai, Mami Ogita, Hideomi Yamashita, Atsuto Katano, Keiichi Nakagawa

**Affiliations:** 1 *Department of Radiology, MD, the University of Tokyo Hospital, Tokyo, Japan. *; 2 *Graduate Schools for Law and Politics, MA, the University of Tokyo, Tokyo, Japan. *; 3 *Department of Radiology, MD, PhD, the University of Tokyo Hospital, Tokyo, Japan. *; 4 *Department of Comprehensive radiation oncology, MD, PhD, the University of Tokyo, Tokyo, Japan. *

**Keywords:** Radiotherapy, cancer survivors, decision making, patient satisfaction, second opinion, Japan

## Abstract

**Objective:**

The radiotherapy utilization rate in Japan is lower than that in other developed countries. This study identified factors associated with the low rate, by conducting an online survey of Japanese cancer survivors.

**Methods:**

We reviewed the web survey results of Japanese cancer patients. The survey examined the process of choosing treatments and the actual treatment received. We included respondents whose most engaged-in treatment was either radiotherapy or surgery, dividing them into two groups. We used the chi-square test to compare the patients in the both groups for their impression of the therapy, decision-making approach, and decision to seek second medical opinions (SMOs). To assess the relationship between seeking SMOs, being most engaged in radiotherapy, and feeling satisfied, we used the structural equation modeling (SEM) approach.

**Results:**

We included 139 patients in the radiotherapy group and 681 patients in the surgery group. Compared with patients in the surgery group, more patients in the radiotherapy group sought SMOs (19% vs. 28%), shared opinions with their doctor (27% vs. 42%), and were satisfied with their treatment (69% vs. 78%). SEM analysis showed that seeking SMOs contributed to radiotherapy being the most-engaged-in therapy (β = 0.23; P < 0.01), and the treatment contributed to the satisfaction (β = 0.15; P < 0.01).

**Conclusion:**

Patients who underwent radiotherapy felt more satisfied with the treatment than patients who underwent surgery. Perceptions about radiotherapy and SMOs may be a reason for the low utilization of radiotherapy in Japan.

## Introduction

In Japan, cancer has been the leading cause of death since 1981. The National Cancer Center estimated that a total of 1 017 200 cases of cancers were newly diagnosed, and 380 300 patients died in 2019 (Hori et al., 2015). Although radiotherapy is one of the primary treatment modalities in cancer management, only a quarter of cancer patients in Japan received radiotherapy (Hori et al., 2015; Delaney et al., 2005; Ringborg et al., 2009; Japanese Society for Radiation Oncology Database Committee, 2105). In contrast, in other developed countries such as Europe and the US, approximately 50% of cancer patients were treated with radiotherapy (Delaney et al., 2005). Radiotherapy would become more critical because the number of cancer patients is increasing in Japan, mainly due to an aging population (Hori et al., 2015). The reasons for lower radiotherapy utilization rates in Japan were unclear.

Globally, radiotherapy has been associated with adverse events such as tiredness, distress, depression, and even sleeplessness (Andersen et al., 1984; Chen et al., 2009; Munro et al., 1996; Dhruva et al., 2012). Similarly, in Japan, radiation was perceived to be dreadful and dangerous by non-medical professionals (Matsui 2003; Japan Atomic Energy Relations Organization, 2019). Additionally, radiotherapy was sometimes thought to cause anxiety (Hirota et al., 2005; Shimotsu et al., 2010). Japanese people may have a more negative perception of radiation than people in other countries, partly due to the use of atomic bombs in the Second World War and the Fukushima nuclear accident (Hirota et al., 2005). Negative perceptions of radiotherapy may be one of the reasons for the lower rates of implementation.

Second medical opinions (SMOs) are independent advice given by doctors other than the doctor-in-charge. In Japan, patient referral documents for SMOs were approved by the Ministry of Health, Labour and Welfare (MHLW) under insurance coverage for the first time in 2006, although the practical consultation for SMOs was not covered. SMOs could cause changes in diagnosis and treatment plans and improve patient-physician communications and patients’ satisfaction in cancer patients, but they are not popular in Japan (Mellink et al., 2006; Morrow et al., 2009, Schook et al., 2014; Philip et al., 2010). In 2011, the MHLW reported that 29.6% of out-patients and 45.0% of hospitalized patients hoped for SMOs on their cancers, with only a third of those patients consulting other doctors (The Ministry of Health Labor and Welfare, 2011). A Japanese typical decision-making process, in which the patients leave their entire treatment up to their physicians’ decisions, led to the low frequency of seeking SMOs (Slingsby, 2004; Watanabe et al., 2008). Little evidence is available concerning the association between asking SMOs and being treated with radiotherapy.

We hypothesized that there is an association between the Japanese perception of radiotherapy, decision-making styles, low SMOs rates, and low utilization rates of radiotherapy. We retrospectively analyzed the results of the online survey that had been done to evaluate the utilization rate of radiotherapy and the impact of SMOs on the decision-making process about cancer treatments in Japanese cancer patients.

## Materials and Methods

*Study design and participants*

We analyzed the results of the web survey planned by Varian Medical Systems, K.K. to identify the cause of the low utilization rate of radiotherapy in Japan and analyze how prevalent SMOs were among Japanese cancer patients. This investigation was administered by Macromill Carenet, Inc., an independent market research firm specializing in medical fields. A screening survey was conducted from 15 to 18 December 2017, and a primary survey was done from 11 to 21 June 2018. Macromill Carenet, Inc. holds a “disease panel,” which is a pool of potential medical research participants enrolled on the website. The company registered more than 300,000 patients with their characteristics such as their sex, age, occupation, residence status, marital status, child status, and annual household income.

In the screening survey, eligible participants were members of this “disease panel,” aged 18–99 years, and not working in advertising. Respondents at the screening survey were recruited to the primary study if they had been diagnosed with specific cancers including lung, liver, esophagus, malignant lymphoma, breast, cervical, prostate, or head and neck (H&N) carcinomas, where radiotherapy is used as one of the standard treatments in Japan. If participants had experienced multiple cancers, they were instructed to answer in relation to the latest one. In our analysis, we included participants who had undergone radiation therapy and/or surgery for their cancers and had engaged most in either of the two.

At the screening, participants were asked about their cancer types, stages, treatments, time of diagnosis, and availability of SMOs. In addition to the screening answers, we used four questions related to their impression of the therapy, SMOs, decision-making styles, and satisfaction, from the primary survey for our analysis, although the primary survey contained 25 questionnaires. Throughout the survey, all participants responded to the same questionnaires. The contents of five questions are presented in the supplementary data.

*Statistical analysis*

We used the chi-square test to compare the populations engaged most in radiotherapy (RT group) and surgery (surgery group) and to assess the impressions toward radiotherapy and surgery, the decision-making approach, and utilization of SMOs between the two groups. To measure the strength of the relationship between the decision-making approach and utilization of SMOs, we used Cramer’s V correlation. Cramer’s V takes values between 0 and 1, close to 0 if the relevance is weak and close to 1 if the relevance is strong. To compare the satisfaction with their treatments, we used Student’s t-test. We performed the structural equation modeling (SEM) approach to assess the relations among consulting for SMOs (binary variable), belonging to the RT group (binary variable), and satisfaction with the whole treatment (continuous variable). To assess how well the models fit the data, we used multiple indices; comparative fit index (CFI), goodness-of-fit index (GFI), adjusted goodness-of-fit index (AGFI), root mean square error of approximation (RMSEA), and standardized root mean square residual (SRMR). Values ≥ 0.90 are criteria for a good fit per GFI and AGFI, values ≥ 0.95 indicate a good fit per CFI, and values ≤ 0.05 represent a good fit per RMSEA and SRMR. All statistical analysis was conducted using R (version 4.0.2). A P-value of less than 0.05 was considered statistically significant.

## Results

*Study enrollment*

**Figure 1:**
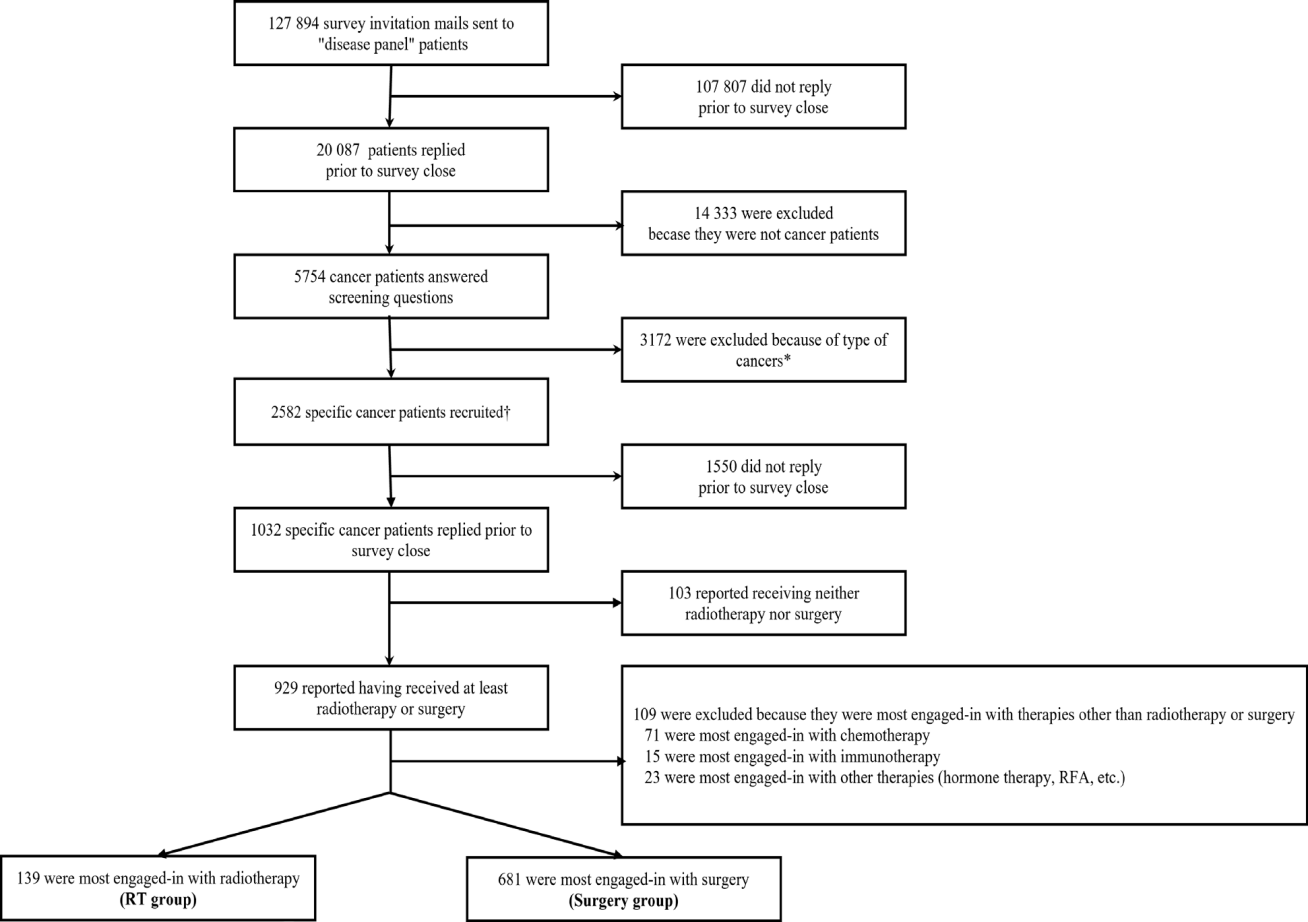
The Flow of Respondents Through Recruiting, Screening, and Completion of the Survey. * Respondents with stomach, colorectal, pancreas, kidney, ureteral, adrenal, biliary tract, bladder, thyroid, skin, uterus, ovarian, vulva, vaginal, testicular, or pediatric cancer; melanoma; multiple myeloma; osteosarcoma; soft tissue sarcoma; or brain tumor were excluded. †The specific cancers are lung, liver, esophagus, malignant lymphoma, breast, cervical, prostate, and head, and neck cancers

The flowcharts of study participants are shown in Figure 1. The response rate to the screening survey was 15.7% (20,087 of 127,894). The eligibility rate of screening was 28.6% (5,754 of 20,087); some of them were excluded because they were not diagnosed with cancer. Of these eligible participants, 2,582 cancer patients qualified for the primary survey, and the rate was 44.9 % (2,582 of 5,754). The questionnaires were completed by 1032 participants prior to the survey close. The response rate of the primary survey was 40.0% (1,032 of 2,582). Participants included in our analysis were divided into two groups: those who answered their most-engaged-in treatments were radiotherapy (RT group, N = 139) or surgery (surgery group, N = 681).

*Study sample characteristics*

The demographic and clinical characteristics are presented in Table 1. The RT group included more men, older patients, and more advanced cancers. Of the RT group, 47% and 29% reported prostate and H&N cancer, respectively. On the contrary, 26% of the patients in the surgery group had cervical cancers, 20% had breast cancers, and 18% had lung cancers. More patients in the surgery group had undergone chemotherapy than those in the RT group (79% vs. 65%; P = 0.001).

*Impressions about radiotherapy and surgery*

**Figure 2:**
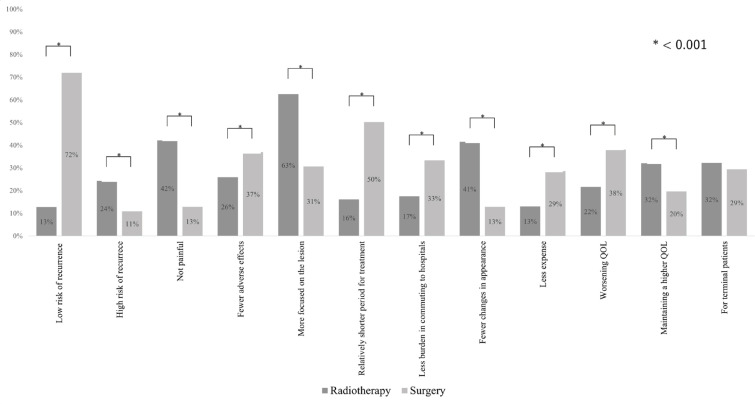
Comparison of the Impressions about Radiotherapy and Surgery in All Participants. QOL, quality of life

Figure 2 shows the difference between impressions toward radiotherapy and surgery. Compared to the participants in the surgery group, more participants in the RT group answered that treatment was not painful (43% vs. 13%; P < 0.001), and caused fewer changes in appearance (41% vs. 13%; P < 0.001). In contrast, we observed many negative impressions of radiotherapy. Compared to the participants in the surgery group, fewer participants in the RT group answered that treatment resulted in a low risk of recurrence (13% vs. 72%; P < 0.001), caused fewer adverse effects (26% vs. 37%, P < 0.001), and had less expense (13% vs. 29%, P < 0.001). There was no significant difference in the reply to the question of whether or not the treatment was for terminal patients (RT group vs. surgery group, 32% vs. 29%; P = 0.24).

*SMOs and decision-making approach*

Participants in the RT group answered that they were more likely to have obtained SMOs than those in the surgery group (28% vs. 19%; P = 0.010), as shown in Table 2A. Table 2B represents the decision-making approach of participants toward their cancer treatment choices. Similar to seeking SMOs, RT group participants were more likely to select the shared approach (share opinions and discuss treatment options with the doctor) than surgery group participants (42% vs. 27%; P < 0.001). There was a weak relationship between the participants’ answers to decision making and SMOs (P < 0.001, Cramer’s V = 0.14). For each approach, there was a stronger relationship between decision making and SMOs in the RT group (P < 0.001, V = 0.31) than in the surgery group (P < 0.001, V = 0.13). In the RT group, patients who answered the shared approach obtained SMOs more frequently than those who answered other approaches, although not significantly (37% (22/59) vs. 21% (17/80) ; P = 0.059).

*Satisfaction with the treatment*

As shown in Table 3, more patients in the RT group answered felt more than satisfied than those in the surgery group; 78% of the participants in the RT group were satisfied with their whole treatment, whereas the rate was 69% among the surgery group (P = 0.003). The structural equation modeling approach to analyze the relationship among seeking SMOs, choosing radiotherapy as the treatment most-engaged in, and feeling satisfied with the course of the whole treatment yielded a good fit (CFI = 1.00, GFI = 0.998, AGFI = 0.992, RMSEA = 0.000, SRMR = 0.000). This shows that the model gives an acceptable representation of the data. Consulting for SMOs had a direct effect on answering radiotherapy as the treatment most-engaged-in (β = 0.232). Selecting radiation treatment as the most engaged-in treatment also contributed positively to satisfaction (β = 0.151). All coefficients were statistically significant (P < 0.01).

**Table 1:** Demographic and Clinical Characteristics of Radiotherapy and Surgery Groups

		Treatment group				
		Radiotherapy (N=139)		Surgery (N=681)		
		N	%	N	%	p-value
Gender	Male	119	86%	327	48%	< 0.001
	Female	20	14%	354	52%	
Age group (year)	≤49	13	9%	149	22%	< 0.001
	50-59	19	14%	192	28%	
	60-69	47	34%	194	28%	
	70-79	49	35%	127	19%	
	≥80	11	8%	19	3%	
Marital status	Single/widowed/divorced	18	13%	169	25%	0.003
	Married	121	87%	512	75%	
Number of children	Zero	36	26%	199	29%	0.492
	≥1	103	74%	482	71%	
Employment	Government worker	2	1%	20	3%	< 0.001
	Manager	4	3%	15	2%	
	Office Worker	23	17%	128	19%	
	Self-employed	5	4%	36	5%	
	Work-Part time	14	10%	103	15%	
	Stay at home	4	3%	140	21%	
	Unemployed	70	50%	185	27%	
	Other/Unknown	17	12%	54	8%	
Income level	<JPY 4 000 000	56	40%	263	39%	0.199
	≥JPY 4 000 000	74	53%	335	49%	
	Unknown/no answer	9	6%	83	12%	
Cancer type	Lung	10	7%	122	18%	< 0.001
	Liver	5	4%	48	7%	
	Esophagus	2	1%	55	8%	
	Malignant lymphoma	4	3%	13	2%	
	Breast	7	5%	139	20%	
	Cervix	6	4%	174	26%	
	Prostate	65	47%	79	12%	
	H&N	40	29%	51	7%	
Cancer stage	0-I	37	27%	371	54%	< 0.001
	II	42	30%	123	18%	
	III	30	22%	89	13%	
	IV	17	12%	33	5%	
	Unknown	13	9%	65	10%	
Chemotherapy	Yes	49	35%	145	21%	0.001
	No/Unknown	90	65%	536	79%	
Radiotherapy	Yes	139	100%	134	20%	< 0.001
	No/Unknown	0	0%	547	80%	
Surgery	Yes	20	14%	681	100%	< 0.001
	No/Unknown	119	86%	0	0%	

Data are presented as the number of subjects in each group with percentages.; Abbreviation: JPY, Japanese Yen; H&N, head and neck.

**Table 2A:** Comparison of Utilization of SMOs between Radiotherapy and Surgery Groups

	Treatment group				
	Radiotherapy (N=139)		Surgery (N=681)		
	N	%	N	%	p-value
I have sought SMOs	39	28%	129	19%	0.01
I knew about the system of SMOs but did not use it	44	32%	195	29%	
I knew about the system of SMOs but did not know how to use it	47	34%	316	46%	
I have never heard of SMOs	9	6%	41	6%	

Data are presented as the number of subjects in each group with percentages; SMOs, Second medical opinions

**Table 2B:** Comparison of Decision-Making Approach between Radiotherapy and Surgery Groups

	Treatment group				
	Radiotherapy (N=139)		Surgery (N=681)		
	N	%	N	%	p-value
Shared approach	59	43%	184	27%	<0.001
Informed approach	33	24%	275	40%	
Paternalistic approach	45	32%	203	30%	
None of the above	2	1%	19	3%	

Data are presented as the number of subjects in each group with percentages.

**Table 3:** Comparison of Satisfaction Through Whole Treatments between Radiotherapy and Surgery Groups

	Treatment group				
	Radiotherapy (N=139)		Surgery (N=681)		
	N	%	N	%	p-value
Felt more than satisfied	41	30%	112	17%	0.003
Felt satisfied	67	48%	356	52%	
Felt neither satisfied nor dissatisfied	25	18%	179	26%	
Felt dissatisfied	4	3%	18	3%	
Felt more than dissatisfied and should have selected other treatments	2	1%	16	2%	

Data are presented as the number of subjects in each group with percentages.

## Discussion

This study investigated the impression of radiotherapy and the impact of SMOs on choices and satisfactions of cancer treatments among Japanese cancer patients. Radiation therapy was considered to be less painful and helped maintain a higher quality of life, but it was not considered to be a radical way of treatment. Patients with an attitude toward the shared approach tended to obtain SMOs, and obtaining SMOs had a positive impact on receiving radiotherapy, resulting in improved satisfaction among cancer patients.

Previous research clarified that most cancer patients who underwent radiotherapy felt it caused anxiety (Andersen et al., 1984; Chen et al., 2009; Munro et al., 1996; Dhruva et al., 2012; Hirota et al., 2005; Shimotsu et al., 2010). In 2005, the Japanese Society for Radiation Oncology reported that patients thought radiotherapy had both strong effects and strong side-effects (46%), radiotherapy was somehow scary (28%), and it was unfamiliar (10%) (Hirota et al., 2005). In our analysis, radiation therapy was considered less effective than surgery but was not considered to worsen the quality of life. Although our result was not consistent with the previous research, the impression was ambiguous and should be considered relative rather than absolute. If a radical cure is the highest priority among cancer patients and the survey participants thought radiotherapy would be inferior to surgery in therapeutic effects, our study supported the possibility that the negative impressions toward treatment itself would be one reason for the infrequent use of radiotherapy in Japan. Further research is needed on whether or not the patients’ impression of treatment is related to treatment selection. If it is correct, improving the image of radiation therapy in Japan may increase its prevalence.

Former studies have shown that factors motivating patients to seek SMOs were as follows: dissatisfaction with an explanation from the first doctor; the information provided and the way it was provided; involvement in decision making; hope for other opinions; and reassurance of diagnosis and treatment recommendations (Philip et al., 2010; Ruetters et al., 2016; Lund et al., 2009; Tattersall et al,2009). Besides, some studies reported that reasons to avoid SMOs were ignorance of the existence of SMOs, misconceptions about the purpose of SMOs, and anxiety due to information overload (Ruetters et al., 2016; Denberg et al., 2006). Moreover, a study of breast cancer patients implicated that dissatisfaction with the first consultation was related to seeking SMOs, and apprehension of SMOs was related to their treatment choices and that the series of their treatments were related to their satisfaction (Lund et al., 2009). Another small observational study showed that patients with gynecologic cancer undergoing radiotherapies were more likely to seek SMOs (Tam et al., 2005). Consistent with the past research, we observed that in the shared-approach group, participants who are likely to engage in decision making, were more likely to seek SMOs, and this tendency was more evident among patients in the RT group. Furthermore, we observed a positive association between seeking SMOs, belonging to the RT group, and improving total treatment satisfaction using an SEM analysis. We did not have enough data to explain the reasons for this satisfaction after the whole therapy. We speculated those reasons as follows; radiation therapy itself may be associated with the higher satisfaction; a feeling of seeking SMOs and getting more closely involved in selecting treatment methods may improve satisfaction; if the survey patients give importance to their side-effects more than therapeutic effects after completing therapy, they might view radiotherapy more favorably because of the impressions of fewer side-effects as shown in Figure 2. Throughout our analysis, we found the possibility that consulting for SMOs would influence receiving radiotherapy. This means that a lower prevalence of SMOs could be another reason for fewer radiation treatments in Japan.

Our research has several limitations that must be kept in mind. 1) This was a cross-sectional survey, not a longitudinal one. 2) The validity and reliability of the questionnaires of this survey have not been evaluated. 3) Due to the characteristics of web surveys, the participants did not represent cancer patients in Japan; they might have acquired higher internet-literacy. 4) The screening response rate was as low as 15.7%, and the response rate of the primary survey was 40.0%. Each rate was not high but would be acceptable, because even a response rate below 10% was not uncommon for web surveys (Mol, 2017). 5) Patients who have worsened in health since completing their treatments would be less likely to participate in this survey than healthy patients. This might lead to a sample selection bias. 6) The medical characteristics, such as clinical situation and prognosis, were not asked. We could not assess any impact of those. 7) The participants’ responses might have been influenced by the received treatments. They might have thought their therapies were more favorable than others. This might result in a recall bias. 8) There was a significant difference in the characteristics between the two groups, including the type and stage of cancer. We could not adjust because of the number of patients. Since this study was about decision-making and satisfaction rather than prognosis, the impact of those differences is considered to be relatively small. 9) Most importantly, the division of the two groups was attributed to question one (What was your treatment you engaged in most for your cancer?). This uncommon classification could lead to difficulty in interpreting the result of the study. Despite these limitations, we believe our findings suggest the significance of individual characteristics in explaining the low utilization of radiotherapy in Japan.

In this study, we considered two possible psychological reasons for the lower prevalence of radiotherapy in Japan. It is known that decision-making factors in oncology include decision-makers’ characteristics and decision-specific characteristics (Glatzer et al., 2020). Our results would indicate the individual characteristics. This survey revealed some differences in cancer patients’ impression of radiotherapy and surgery; it also revealed the relations among seeking SMOs, receiving radiation therapy, and improving satisfaction with the whole treatment. 

In conclusion, our study indicates that the low utilization of radiotherapy in Japan may be related to perceptions about radiotherapy and SMOs. We believe that evaluating the current Japanese awareness of radiotherapy and educating the public correctly would lead to the proper utilization of radiotherapy.

## Author Contribution Statement

The authors confirm contribution to the paper as follows: study conception: Masanari Minamitani, Tomoya Mukai, and Mami Ogita; analysis: Masanari Minamitani, Tomoya Mukai; interpretation of results:Masanari Minamitani and Keiichi Nakagawa; draft manuscript preparation: Masanari Minamitani, Hideomi Yamashita, and Atsuto Katano. All authors reviewed the results and approved the final version of the manuscript.
